# Patient-centered development of clinical outcome assessments in early Parkinson disease: key priorities and advances

**DOI:** 10.1038/s41531-024-00716-z

**Published:** 2024-05-14

**Authors:** Tiago A. Mestre, Glenn T. Stebbins, Diane Stephenson, David Dexter, Karen K. Lee, Yuge Xiao, Tien Dam, Catherine M. Kopil, Tanya Simuni

**Affiliations:** 1 Ottawa Hospital Research Institute; University of Ottawa Brain and Mind Research Institute; Division of Neurology, Department of Medicine, University of Ottawa, The Ottawa Hospital Ottawa, Ottawa, ON Canada; 2 Department of Neurological Sciences, Rush University Medical Center, Chicago, IL USA; 3 Critical Path Institute, CAMD, Tucson, AZ USA; 4 Parkinson’s UK, London, UK; 5 Parkinson Canada, Toronto, ON Canada; 6 The Michael J. Fox Foundation for Parkinson’s Research, New York, NY USA; 7 Neumora Therapeutics, Cambridge, MA USA; 8 Northwestern University Feinberg School of Medicine, Chicago, IL USA

**Keywords:** Parkinson's disease, Drug therapy

## Abstract

Novel therapies with the ability to delay disease progression are a gap in the care of people living with Parkinson disease (PD) today. Clinical outcomes assessments (COAs) that are sensitive to the earliest clinical changes in PD are deemed essential for a successful therapeutic development. To understand the current landscape of COAs use in clinical trials in PD and define priorities for future research in the field, a stakeholder roundtable meeting was held in November 2022. The current paper 1) proposes the collaborative development of patient-centric COAs that can adequately document the effectiveness of disease modification therapies in PD based on key priorities identified during this initial meeting, 2) summarizes the progress made in the subsequent 12 months, and 3) presents the deliverables expected in the near future. Key priorities include 1) the development of a consensus conceptual model of early PD experiences, 2) the adaptation of existing patient-reported outcomes (PROs), 3) the investigation of the role of observer-reported outcomes in addition to 4) enabling diversity in PD research and advocacy, 5) fostering data sharing, and 6) reaching consensus on a biological staging system for PD to drive the development of appropriate PROs for biologically defined populations.

## Introduction

Parkinson disease (PD) is a progressive neurodegenerative disorder with a societal burden expected to increase rapidly. In some countries, PD incidence could double in the next 50 years^1^. It is well-established that in the years preceding the emergence of parkinsonian features that currently enable a clinical diagnosis of PD^2^, various motor and non-motor manifestations such as REM sleep behavior disorder (RBD), hyposmia or constipation^3^ become apparent as an expression of the underlying biology. Although dopaminergic therapy changed dramatically the natural history of the disease and improved the quality of life of those living with clinical PD, these symptomatic treatments are often associated with side effects such as motor complications, and their effectiveness can be reduced over time (or become ineffective) for some features of PD, such as gait and balance. In addition, there is a lack of effective therapies for many non-motor features of PD. There is no therapy able to slow down, halt, or reverse PD progression. In the last 12 months, the field has converged on the urgent need to develop a biological definition and staging/classification of PD to enable therapeutic intervention studies that target the relevant biological process(es), ideally before the earliest clinical signs of parkinsonism^4,5^. Biologically targeted therapies are expected to delay the emergence of key disease milestones such as the presence of diagnostic motor features, treatment-associated motor complications, postural instability, dysautonomia, or dementia^6^. A direct implication of being able to diagnose the underlying biology before the emergence of a diagnostic parkinsonism is the need to measure the earliest clinical changes to evaluate the impact of therapeutic interventions earlier in the disease using outcomes that are relevant to those with a lived experience. In summary, regulatory-accepted clinical outcome assessments (COAs) sensitive to clinical changes in early biological disease are urgently needed.

Historically, COAs have been developed by independent investigators, oftentimes academic researchers. However, there is a growing need to collaborate with diverse partners, including regulatory agencies, to enable efficiencies for the many clinical trials emerging in PD, particularly with a focus on early disease intervention. Patient-focused drug development has emerged as a key priority of regulatory agencies around the world, and calls for a systematic approach to ensure that patients’ experiences, perspectives, needs, and priorities are captured and incorporated into drug development in a meaningful manner^7^. To understand the current landscape of COAs used in clinical trials in PD and develop a roadmap for the development of patient-centric COAs, a stakeholder roundtable meeting was held in November 2022 hosted by The Michael J. Fox Foundation for Parkinson’s Research (MJFF), Parkinson’s UK, and Parkinson Canada and Critical Path Institute bringing representatives from academia and industry together, in addition to representatives of regulatory agencies, community partners, patient advocates, and research funders. While a detailed summary of the 2022 PD Endpoints Roundtable meeting was published^8^, the current manuscript provides an updated and in-depth perspective of the main findings of this meeting in relation to COAs, presents the progress made in the 12 months following the 2022 meeting and its near-term deliverables. Examples in the Huntington’s disease (HD) and Alzheimer’s disease (AD) fields are showcased to highlight the successes of aligning COA development with a biological definition, staging or classification of a neurodegenerative disease.

## Current landscape of available COAs in PD

Clinical outcome assessments (COAs) are measures that describe or reflect how a patient feels, functions, or survives. As such, COAs are essential for measuring disease in a clinically meaningful way^9^, and they are classified according to the source of clinical information^9^ into patient-reported outcomes (PROs), observer-reported outcomes (ObsROs), clinician-reported outcomes (ClinROs) and performance-based outcome (PerfOs). In the context of COAs, PROs are unique instruments that incorporate the patient’s voice directly. A relatively limited number of PROs have been developed for PD (e.g., The Parkinson’s Disease Questionnaire-39^10^, Parkinson’s Disease Activities of Daily Living, Interference, and Dependence^11^).

Numerous standardized measurement tools have been used for clinical research in the field of PD, namely, in clinical trials (Table 1). The Unified Parkinson’s Disease Rating Scale (UPDRS) and its revised version, the Movement Disorders Society sponsored revision of the Unified Parkinson’s Disease Rating Scale (MDS-UPDRS)^12^ are the most frequently used COAs in PD, constituting the current gold standard of a comprehensive clinical measurement. The sections of the MDS-UPDRS include both ClinROs (Parts Ia, III, and IV) and PROs (Parts Ib and II). The revised version was developed to address limitations of the original UPDRS, namely the inability to optimally capture the earliest phases of clinical PD and the lack of patient input in item development. Examples are the inclusion of a ‘slight’ response option and of novel items such as “Fatigue”, “Doing hobbies and other activities” and “Getting out of bed, car, or deep chair” to fully capture the experience of people living with clinical PD^13^.

**Table. 1:** Clinical outcomes assessments used clinical trials in early clinical Parkinson disease currently registered in clinicaltrials.gov (*n* = 142, adapted from ref. ^8^)

Patient- or clinician-reported outcome scales	Number of trials
Movement Disorder Society-Unified Parkinson’s Disease Rating Scale (MDS-UPDRS)	
Part I	25
Part II	44
Part III	58
Part IV	14
Parts II and III	30
Parts I–III	4
Summary Score	48
Parkinson’s Disease Questionnaire	37
Clinical Global Impression Scale	
Patient Global Impression Scale-Disease Improvement	13
Clinical Global Impressions-Improvement Scale	24
Clinical Global Impressions-Change Scale	2
Clinical Global Impressions-Severity Scale	6
Clinical Global Impression Scale Summary Score	2
Epworth Sleepiness Scale	11
Non-Motor Symptoms Scale for Parkinson’s Disease	7
Hoehn and Yahr Scale	17
Schwab and England Activities of Daily Living Scale	14
Visual Analog Scale: Pain	4
Columbia Suicide Severity Rating Scale	4
ON/OFF	
ON	16
OFF	10
Parkinson’s Disease Fatigue Scale	2
Questionnaire for Impulsive-Compulsive Disorders in Parkinson’s Disease Rating scale	5
Performance-based measures	
Montreal cognitive assessment	12
Timed up-and-go	8
Total	520

Despite the attempt to assess the mildest manifestations of PD, clinimetric gaps have been observed for the MDS-UPDRS Parts Ib and II in people with a recent diagnosis of PD (less than 1 year), namely, a significant floor effect, suboptimal scale-to-sample targeting with item coverage deficit for milder representations in the continuum of motor sign severity (Part III) or the impact of motor features in experiences of daily living (Part II)^14^, in addition to disordered thresholds for some items of the MDS-UPDRS part II^14^. These findings highlight the need for a sensitive, reliable, and meaningful COA that can be used as an efficacy outcome measure in diseasemodification or prevention clinical trials.

## Areas of growth for successful development of COAs in early PD

### Definition of concept of interest and context of use in early clinical PD

We propose that early clinical PD corresponds to the earliest clinical manifestations, either the mildest signs of parkinsonism or non-motor manifestations of the underlying synucleinopathy (examples are RBD, hyposmia, or constipation), that are specific and relevant to PD. This concept provides an operational definition of a stage in the natural history of PD that would allow the participation of individuals in clinical trials much earlier than the current practice for PD therapeutic development. Yet, we recognize that this definition may not be early enough in the course of the biological disease process. The field needs to transition to a PD biological definition that will reflect the biological substrate of the disease even before the earliest clinical manifestations, which will enable disease modification trials to consider preclinical stages and the adoption of “prevention and/or delayed transition to a clinical stage” as a possible efficacy outcome.

At the 2022 meeting, the idea of developing a biological staging system was introduced. A well-defined staging scheme composed of consecutive discrete periods in the natural history of PD and defined through precise quantitative criteria is an urgent need for drug development in PD^4^. Two proposals have been reported since then^4,5^. A major impact of a biological definition and consensus staging/classification system of PD is COA development, as validation of outcomes measures is population- and context-specific. Consequently, the future development of COAs will need to be stage-specific in individuals meeting the criteria of a valid biological PD definition.

Although existing COAs have attempted to capture the earliest changes in clinical PD, traditionally with reference to a clinical diagnosis, there is no single COA that can adequately capture both the earliest motor features and the well-established non-motor stages in PD predating the onset of a diagnostic parkinsonism.

With the proposed definition of early clinical PD, COAs that comprehensively cover the earliest clinical changes in people living with PD need to meet benchmarks of development and validation as defined by regulatory agencies^15^, namely, patient-centredness. To address this fundamental gap, a concept of interest (COI) adequate to “early clinical PD” as defined above is warranted, together with a context of use (COU) need to be appropriately defined as foundational stepping stones of a COA. A COI corresponds to the aspect of an individual’s clinical, biological, physical, or functional state or experience that a COA is intended to capture or reflect^7^. For example, a COI applicable to early clinical PD needs to capture the most significant aspects of the lived experience of people before a clinical PD diagnosis, in addition to those with a recently established clinical diagnosis^2^. A COU establishes the “purpose and conditions”, in which it is valid to use a clinical measurement^7^. The use of COAs in clinical trials of potential disease modification therapies (DMTs) in early PD is a priority. Consequently, a COA used as a primary endpoint for a novel intervention with a disease modification intent in early clinical PD is expected to capture a meaningful change at this disease stage during the time course of a clinical trial, and the response options need to reflect distinguishable and sensitive changes in severity and impairment.

### Incorporation of the patient voice in COA development in early clinical PD

The development of patient-centric endpoints is rooted in the ability to document the patient’s perspective in a comprehensive and meaningful way, something that was emphasized by people living with PD during the meeting. Patient-focused drug development is a growing priority for regulatory agencies, including the US Food and Drug Administration (FDA), that have issued guidances on collecting comprehensive and representative input to a pre-defined COI and COU^16^ and selecting appropriate methodologies for a target population, study characteristics, or study objectives^17^. In the 2022 PD Endpoints Roundtable meeting, various partners presented on independent COA development programs targeting an “early PD” population showcasing a diverse use of methodologies and approaches to COA development (Table 2). The ensuing discussion by the different partners during the meeting led to the conclusion that joint collaborative efforts (academia, industry, and patients/care partner representatives, regulatory agencies) in the pre-competitive space would enhance the successful development of novel COAs in early clinical PD. The collaborative incorporation of different perspectives into scale development, independent of the success of a particular project or drug development program, is expected to increase its acceptability and promote a more generalized use.

**Table. 2:** Case studies of patient-reported clinical outcome assessments in development for early clinical Parkinson disease (PD). PRO - patient-reported outcome

Project	Presenters (affiliation)	Aim	Highlights	Current status (Jan 2024)
Patient Perspectives Informing a Performance Outcome in Early PD	J. Mammen (University of Massachusetts) and J. Adams (University of Rochester) on behalf of CPP 3DT initiative	Incorporation of patient voice in the “Wearable Assessments in the Clinic and Home in Parkinson’s Disease - WATCH-PD” program.	Collection of quantitative and qualitative data in the same protocol. Development of a personal symptom map with identification of “important” vs. “bothersome symptoms.	Study results published^38,39^.
Parkinson’s Disease Motor-Related Impacts Questionnaire	E. Davies and D. Trundell (Roche)	Address the current limitations of the MDS-UPDRS in relation to early clinical PD.	Patient-Centered Conceptual Model of Symptoms. Multi-source iterative approach to collecting patient voice.	Ongoing evaluation of two scales (PD-MIQ and PD-SyQ) to achieve desired sensitivity to measure earliest features of clinical PD.
Novel Patient-Related Outcome Development for Early Parkinson’s Disease	T. Morel (UCB)	New PROs of motor and non-motor symptoms in early clinical PD.	Multi-source iterative approach to collecting patient voice. Conceptual model indicates cardinal motor (mobility, tremor, and functional slowness) and non-motor (pain, sleep, fatigue, and anxiety). Collaboration with patient associations. Strategies to increase cultural diversity.	Clinimetric testing of two motor scales (Functional slowness PRO and Mobility PRO).
Early PD-PRO	T. A. Mestre (University of Ottawa) and G. Stebbins (Rush University)	Develop a new PRO applicable to the peri-diagnostic period in PD (prodromal subjects and recently diagnosed subjects).	Multi-source approach to collecting patient voice. Mapping of lived experience of at-risk groups (RBD and hyposmia). Strategies to increase cultural diversity.	Cognitive pretesting of Early PD-PRO to capture motor and non-motor changes in prodromal and early clinical PD subjects.
Health Measures (started in 2023)	Sara Shaunfield and David Cella (Northwestern University)	Develop a new PRO based on a pre-existing item bank (PROMIS/Neuro-QoL) applied to early PD.	Mapping of a consensus conceptual model and WATCH-PD data to a pre-existing item bank.	Literature review and key stakeholder input completed to prioritize symptom domains.

**Table. 3:** Proposed strategies to expedite patient-centered clinical outcome assessment development in early Parkinson disease (PD)

Areas for growth	Key priorities		Progress in 2023	Goals for 2024
	Theme	Aim		
Concept of interest and context of use	Consensus conceptual model of early PD	Provide a standardized, comprehensive mapping of the earliest clinical lived experience in PD.	First proposal.	Harmonization and finalization.
	Consensus on a biological staging approach in early PD	Align COA development with a consensual classification scheme of PD that will drive research and therapeutic development.	Exploration of cut-offs for clinical stages using the MDS-UPDRS (NSD-ISS system).	Validation of conceptual model in biologically defined individuals (NSD-ISS system). Need to expand to other clinical populations (Dementia with Lewy body). Work towards stage-dependent clinical anchors and COAs (NSD-ISS system).
	Adapt existing PROs	Modify currently available PROs to be used in clinical studies in the short-term.	Construct validity of different combinations of MDS-UPDRS parts to incorporate patient voice.	Abbreviated version of the current MDS-UPDRS most sensitive to early clinical disease.
	Explore the role of ObsRO	Determine the contribution of the knowledge informant to report on the earliest clinical changes in PD.	Expansion of data collection to understand the potential for an ObsRO.	Synthesis of data to develop a Knowledgeable Informant Questionnaire.
	Increase diversity in PD research and advocacy	Develop COA that can be validly used across the PD population.	Research to gather perspectives from underrepresented populations.	Patient organizations to develop processes for supporting researcher engagement with representative volunteers in parallel with expanding diversity of their community of research advocates.
Incorporation of the patient voice in COA development in Early PD	Data sharing of qualitative data	Enable data integration to increase the power and completeness of qualitative research in PD for COA development.	Identify stakeholders and discuss a minimally viable solution.	Define a collaborative action plan for data sharing.

*NSD-ISS* neuronal synuclein disease-integrated staging system, *COA* clinical outcome assessment. *PRO* patient-reported outcome, *MDS-UPDRS* Movement Disorders Society sponsored revision of the Unified Parkinson’s Disease Rating Scale

### Key priorities and progress for fit-for-purpose patient-centered COAs in early clinical PD (Table 3)

The 2022 PD Endpoints Roundtable meeting established a list of priorities and action items for COA development in “early PD”, after half-day workshops and a plenary session. Their progress was presented in a follow-up meeting that took place virtually on 5–6 December 2023, with a similar group of stakeholders. The delivery of this action plan is predicated on different partners coming together around the common purpose of expedited development of COAs in “early PD” in a pre-competitive space.

#### Consensus conceptual model of early clinical PD

A conceptual model provides an easy-to-understand graphic representation of the relationships between clinical features (domains, sub-items) associated with disease and their impacts^18^. A conceptual model is an integral part of developing a COA as it becomes a reference to determine how adequate a scale is in measuring what it proposes to measure^15^. A conceptual model in early clinical PD would provide a structured depiction of significant experiences organized into symptom or functional domains and their corresponding sub-items. Development of this consensus conceptual model requires synthesis and critical appraisal of all available information sources from qualitative research to cohort studies aiming to characterize this stage of PD. This collaborative project started in 2023, collecting and evaluating the best evidence available for patient experiences in early clinical PD with the consultation of various partners, including patient advocates, academia, industry, and the regulatory perspective. The emerging consensus conceptual model will solicit public review/critique and, once this initial model is finalized, will expedite the development of new COAs and inform the revision of existing scales. Future iterations of the model are expected as knowledge evolves.

#### Consensus on a biological definition and staging approach in PD

The use of biological markers that identify those individuals with PD who present with subtle clinical features is pivotal for COA development in “early PD”. Various efforts are underway to define PD stages based on the underlying biology (α-synuclein pathology as measured today by positive α-synuclein aggregation in a seed amplification assay and dopaminergic denervation as measured today by DAT scan)^4^, or providing a classification framework based on various biomarkers^5^. In recent years, regulatory agencies have opened the way for accelerated approval of drugs for seriously disabling disorders based on the use of likely surrogate markers shown to be able to document a disease-relevant modification of biology and the use of intermediate clinical endpoints to document clinical benefit^19^. This has been the case in early AD. The PD field is not ready for surrogate biomarkers yet, but the ability to detect a therapeutic benefit in stages of disease defined biologically requires the aligned development of COAs, starting with an appropriate COU and COI.

#### Adapt existing COAs for use in early clinical PD in the short-term

The modification of existing scales has been recognized by regulatory agencies as a pathway to pragmatically expedite the use of fit-for-purpose COAs in the “early-PD” space for current drug development^7^. For example, the MDS-UPDRS is a clinimetrically robust COA^12^ that has the potential to be revised specifically for early clinical PD populations, namely, by identifying items that are more sensitive to the earliest clinical features of the disease through the use of innovative statistical methodology such as longitudinal item-response theory modeling in currently available datasets of prodromal and early clinical PD populations^20^. This approach is currently ongoing and may address the urgent needs of upcoming disease modification trials in early clinical PD, while novel fit-for-purpose COAs are not yet available. The success of this approach is predicated on the data sharing of appropriate datasets, specifically from contemporary completed trials.

#### Explore the role of ObsRO in early clinical PD

It is reasonable to consider that those sharing the daily lives of people living with PD are well-positioned to report on the earliest changes from normal self. The use of a knowledgeable informant as a source of information for a COA would constitute an ObsRO with the potential to provide a unique perspective on the earliest clinical changes in PD and their impact that is complementary to a PRO. A valid ObsRO rooted in the experience of a knowledgeable informant could be used as co-primary or secondary outcome in future prevention trials once its clinimetric performance is evaluated.

#### Increase diversity in PD research and advocacy

As with other fields, there is an increased awareness that certain racial, ethnic, and socioeconomic groups are underrepresented in PD research^21^. COA development is context-specific and thus requires an equitable representation of the targeted population. The disparity of participation of non-white people with PD in the development of a COA may undermine the ability to capture a robust therapeutic effect due to cross-cultural validity issues. The implementation of recruitment strategies that ensure a diverse representation is required during COA development^22^. This action is challenging, but several initiatives are underway in PD research^23,24^.

#### Data sharing

Data sharing is foundational to the success of COA development. An immediate and achievable goal is the sharing of qualitative data and related pre-publication reports. Qualitative research and the use of mixed methods approaches are standard methodologies to collect the patient voice in COA development. As outlined earlier in this paper (Table 2), several such efforts are now underway. The projects presented during the 2022 meeting illustrate the diversity in the development of novel COAs in early clinical PD supported by academia, funding organizations, and industry. These programs were at different stages of development and included the final results of a patient-centric evaluation of the relevance and impact of outcomes of a digital health technology in individuals with a recent clinical PD diagnosis (PIs: Mammen/Adams), the definition of COA and COU in a patient-centric manner in prodromal and motor clinical PD populations (PIs: Mestre/Stebbins) and the reporting of a conceptual model and item bank development of a new scale in individuals with a recent clinical PD diagnosis (PIs: Morel/UCB, and Trundell and Davies/Roche). Since then, these programs advanced significantly, and a new PRO development project has started (Table 3) documenting the dynamism of the PRO field in early clinical PD and the pressing need for novel measures.

Inspired by examples of collaborative data sharing in the clinical trial arena under the auspice of Critical Path Institute (CPP)^25,26^, there was a consensus that an open data repository is pivotal for synthesizing different sources of data generated in qualitative studies conducted with the aim of capturing the lived experience of subjects in the “early PD” stage (with or without motor features), including (but not exclusive to) focus groups/individual interview data and social media. The breadth and quality of data collected in the projects presented at the 2022 meeting offered a unique opportunity to create the foundations of a qualitative data repository. The ability to access more granular data than the one usually published in scientific reports would open a unique opportunity to increase the overall power of qualitative research that provides deeper and more comprehensive insight, avoids redundancy, and, ultimately, informs a more accurate conceptual model of “early clinical PD” according to the patient lived experience. Since the 2022 meeting, a group started to discuss the framework of this repository.

The field of PD has a history of collaboration and open science driving research. The Parkinson Study Group^27^ or the Parkinson’s Progression Markers Initiative^28^ are examples that paved the way to the development of novel therapies and discoveries on the biology of PD, respectively. The Parkinson Disease Patient Report of Problems (PD-PROP) is a recent example of data sharing applicable to the “early PD” space^29^. An analogous approach is recommended for qualitative data in the context of COA development. A complementary initiative that is pivotal to COA development in early PD is the creation of a master library of symptom domains together with a corresponding item bank to enable the development of adaptive scales using item-response theory that may prove useful to address the variable patient experience before (and soon after) the emergence of diagnostic clinical features.

### A 2024 action plan for fit-for-purpose patient-centered COAs in early PD (Table 3)

The enthusiasm around the accomplishments reported earlier and materialized within 12 months of the 2022 PD Endpoints Roundtable meeting was leveraged into an action plan for 2024 (Table 3) that includes a) harmonization and finalization of a consensus conceptual model for “Early PD” and evaluation of its validity in biologically defined individuals, b) an adapted version of the current MDS-UPDRS that is more sensitive to change in early clinical PD and thus more reflective of clinical meaningfulness in this phase of the disease, c) establishing a collaborative action plan for the development of novel PROs in early PD with open science approaches, and d) moving towards stage-dependent COAs in biologically defined individuals.

### Lessons learned from collaborative efforts in other disease areas

In HD, a scale for the earliest functional changes in Huntingtin gene mutation carriers entitled FuRST 2.0 has been recently developed using a patient-focused development^30^ and engaged regulatory agencies early in development through the Critical Path Institute’s Huntington’s Disease Regulatory Science Consortium (HD-RSC)^25^. In parallel, a biological staging system was defined in HD based on the availability of large datasets from observational studies, allowing the identification of the earliest clinical phase of HD using unequivocal and reliable landmarks in individuals that meet the biological definition of HD^31^. A result of these efforts is the use of FuRST 2.0 in clinical trials (e.g., NCT05358717) that are evaluating novel disease-modifying therapies in individuals defined biologically and staged to the earliest clinical phase of the disease^31^.

AD is the most prevalent neurodegenerative disorder, which has recently celebrated exciting times in the therapeutic landscape with novel DMTs being approved by FDA for early AD^32^. The development of COAs sensitive to early disease stages was a decisive contribution to this outcome. Examples include the use of the Clinical Dementia Rating-Sum of Boxes as the primary efficacy outcome in trials of lecanemab^33^ and of the Integrated Alzheimer’s Disease Rating Scale in a Phase 3 trial of donanemab^34^. The Integrated Alzheimer’s Disease Rating Scale was developed based on two pre-existing AD scales (the Alzheimer’s Disease Assessment Scale – Cognitive Subscale 13-item version and the Alzheimer’s Disease Cooperative Study – instrumental activities of daily living scale)^35^ and illustrates the value of adapting existing scales for near-term use of COAs in early PD clinical trials. Another example is the Alzheimer Disease Cooperative Study Preclinical Alzheimer Cognitive Composite (ADCS-PACC) developed as a primary outcome measure in disease modification trials targeting the asymptomatic phase of AD (COU)^36^. The success stories of COA development used in the context of AD clinical trials cannot be dissociated from the use of clinical trial eligibility criteria based on biomarker status. Altogether, this progress supports an urgent need to develop a biological definition and staging/classification of PD akin to the ones developed for AD and HD^37^ that aligns biology with the proposed mechanism of action of an experimental therapeutic intervention.

### Concluding remarks

The availability of therapies that slow disease progression will revolutionize the lives of people living with PD and care delivery in the future. Patient-centered development of COAs is an integral and essential tool to document a robust disease-modifying effect in early PD, alongside the understanding of disease biology that allows matching individual biology with targeted therapies. The series of meetings herein reported signal a tremendous dynamism with multiple complementary efforts converging towards valid PROs for early-stage PD aligned with PD biology in a collaborative framework. A pre-competitive space in which different partners can collaborate is the ideal strategy to yield impact and expedite the development of regulatory acceptable instruments that can be used in first-ever prevention trials in PD.

### Reporting summary

Further information on research design is available in the Nature Research Reporting Summary linked to this article.

### Supplementary information

Reporting summary
